# A cost analysis of introducing an infectious disease specialist-guided antimicrobial stewardship in an area with relatively low prevalence of antimicrobial resistance

**DOI:** 10.1186/s12913-016-1565-5

**Published:** 2016-07-27

**Authors:** Peter Lanbeck, Gunnel Ragnarson Tennvall, Fredrik Resman

**Affiliations:** 1 Infectious Diseases Research Unit, Department of Clinical Sciences, Malmö, Lund University, Rut Lundskogs gata 3, plan 6, 20502 Malmö, Sweden; 2 IHE, The Swedish Institute for Health Economics, PO Box 2127, SE-220 02 Lund, Sweden

**Keywords:** Economics, Costs, Antimicrobial stewardship, Antimicrobial resistance

## Abstract

**Background:**

Antimicrobial stewardship programs have been widely introduced in hospitals as a response to increasing antimicrobial resistance. Although such programs are commonly used, the long-term effects on antimicrobial resistance as well as societal economics are uncertain.

**Methods:**

We performed a cost analysis of an antimicrobial stewardship program introduced in Malmö, Sweden in 20 weeks 2013 compared with a corresponding control period in 2012. All direct costs and opportunity costs related to the stewardship intervention were calculated for both periods. Costs during the stewardship period were directly compared to costs in the control period and extrapolated to a yearly cost. Two main analyses were performed, one including only comparable direct costs (analysis one) and one including comparable direct and opportunity costs (analysis two). An extra analysis including all comparable direct costs including costs related to length of hospital stay (analysis three) was performed, but deemed as unrepresentative.

**Results:**

According to analysis one, the cost per year was SEK 161 990 and in analysis two the cost per year was SEK 5 113. Since the two cohorts were skewed in terms of size and of infection severity as a consequence of the program, and since short-term patient outcomes have been demonstrated to be unchanged by the intervention, the costs pertaining to patient outcomes were not included in the analysis, and we suggest that analysis two provides the most correct cost calculation. In this analysis, the main cost drivers were the physician time and nursing time. A sensitivity analysis of analysis two suggested relatively modest variation under changing assumptions.

**Conclusion:**

The total yearly cost of introducing an infectious disease specialist-guided, audit-based antimicrobial stewardship in a department of internal medicine, including direct costs and opportunity costs, was calculated to be as low as SEK 5 113.

**Electronic supplementary material:**

The online version of this article (doi:10.1186/s12913-016-1565-5) contains supplementary material, which is available to authorized users.

## Background

The emergence of antimicrobial resistance is described by the World Health Organization to be “an increasingly serious threat to global public health that requires action across all government sectors and society” [1]. Several factors contribute to the spread of antimicrobial resistance in the community. The misuse or overuse of antibiotics in human medicine is one such factor, and possibly the factor that has been discussed and addressed the most [2].

The term ‘Antimicrobial stewardship programs’ (ASPs) is an umbrella definition that in practice may encompass several different measures. ASPs have been introduced widely to address the misuse of antibiotics in human medicine [3, 4], and hospital programs may include restrictive measures as well as persuasive interventions including audit and feedback methodology [5]. There is no consensus on what outcomes should be monitored when an ASP is launched, and opinions on what outcomes are most important vary from different perspectives [6]. Unfortunately, there is still no conclusive evidence that a reduction of antibiotic use results in reduced antimicrobial resistance [7]. Due to these uncertainties, it is important to monitor individual patient outcomes during the launch of antimicrobial stewardship programs to make sure that programs do not introduce harm. We also believe that it is necessary to analyse the economic consequences of ASPs, to make sure that decisions to introduce ASPs can be objectively compared with other potential efforts.

The full health-economic consequences of antimicrobial stewardship programs are complex to calculate due to uncertainties in long-term effects on costs and benefits, as well as due to uncertainties in attributable costs and effects of the infection. Recently, systematic approaches to address such health economic evaluations have been suggested [8]. Besides the implementation costs and operational costs of the program itself, it is necessary to include the direct costs of antibiotics, including time for handling and materials as well as costs related to patients outcomes, normally hospital lengths-of-stay. It would be desirable to include a calculation of the societal effects, but such calculations are at best uncertain.

In this investigation, the aim was to study the economic consequences of introducing an individual audit-based ASP in Sweden, a region with a limited prior history of systematic ASPs. The program has been demonstrated to be successful in substantially reducing antibiotic use and maintaining favourable patient outcomes [9]. However, since individual audit and feedback-based programs are generally considered as cost-intensive, and since alternative ways of performing stewardship interventions exist, a follow-up cost analysis was deemed valuable. The rates of Clostridium difficile-associated disease (80 cases per 100,000 individuals) [10] as well as the rates of carriage of resistant bacteria [11] are comparatively low in Sweden, with rates of *E. coli* with extended-spectrum betalactamase production at 5 % and rates of methicillin-resistant isolates among *Staphyloccus aureus* at around 1 %. Since this particular program was introduced during ‘normal’ circumstances, i.e. not in response to an outbreak of resistant bacteria or *Clostridium difficile*, we believe that the results can be generalizable to other regions with low proportions of antimicrobial resistance.

## Methods

### Study setting

The original study [9] was performed in the Department of Internal Medicine, Malmö at Skåne University Hospital in Sweden, a hospital with approximately 1100 beds at two sites. The department is a secondary care unit serving patients in general internal medicine, including mainly elderly patients with one or more chronic underlying condition. The geographical area that the hospital serves has a population of approximately 700 000.

### The antibiotic stewardship intervention study design

This cost analysis is based on a quasi-experimental trial with an historic control of the introduction of an antibiotic stewardship intervention. The intervention was performed in four wards of internal medicine, from April 1 through June 20, 2013 as well as August 26 through October 21, 2013. Considering the variability of influenza seasons, the intervention was not performed during influenza seasons. All patients that received, or were prescribed, antibiotics were audited by an infectious disease-specialist twice weekly. The control group consisted of individuals treated with antibiotics during admission to the same wards in the corresponding time period 2012, when no active intervention was in place. An ATC-code based search of the computerized medical records unambiguously identified all cases that had received antibiotics in the two periods, audited or not. Patients receiving antibiotic prophylaxis only were excluded from the analysis [9]. All audits were performed as real-time discussions, including feedback, between the auditing ID specialist and the ward physician. Discussions concerning approximately 25–30 patients took place on each audit day. In some instances, a brief renewed physical examination was performed. All patients with antibiotics were discussed on each occasion, even though the same patient had been discussed in prior visits. Since the ward doctors generally were responsible for a third of patients in a ward, the time spent for each doctor in the ward was approximately 15–20 min per occasion (the four wards had separate medical staff), including write-ups. Even though the patients in the original study were not randomized, they were well-balanced with regards to age, gender and underlying diseases [9].

### Definition of resource categories and unit costs

The present cost analysis is based on the results from the antimicrobial stewardship program that was introduced in the department of internal medicine in southern Sweden in 2013. The clinical aspects of the study have been evaluated and published. This cost analysis is based on the same data [9].

Resource utilization was estimated for audits four hours per time twice weekly for both the specialist physician from the department of infectious diseases and a physician from the study wards. Resource utilization for nurses was estimated based on an assumption about 18 min for preparation and distribution of each dose of IV antibiotics to the patients (the time was based on an inquiry performed at hospital wards) and 6 total minutes per day for administration of each oral antibiotic (normally two to three doses per day).

The implementation costs of the program were based on the time needed to plan the project and provide written and oral information of the project to colleagues to the involved staff. Costs for physicians were calculated based on information from the Swedish Medical association 2015 [12], while the costs for nurses were calculated based on information from Statistics Sweden regarding average salaries in 2014 for professionals in health care [13]. Wages were adjusted to 2015 price level with a labour cost index from Statistics Sweden for employees in the sector of human health and social work activities [14]. A payroll tax for county councils of 41 % was added [15]. Even though the actual audits at the wards could sometimes be performed in less than four hours, though not measured to the minute, costs were based on four-hour audits on each of the forty audits to apply the most conservative cost estimate.

Antibiotic costs were calculated from official price lists in all cases except for ampicillin where information was collected through personal communication with an administration staff in the Southern health care region. For IV antibiotics a daily cost was estimated based on average doses in the medical charts and unit prices from a local database. This database includes information about drugs where the price has been negotiated between pharmaceutical companies and the Southern health care region [16]. Similarly, the cost of oral antibiotics was estimated from the average doses in the medical charts and unit prices from FASS [17]. Whereas all IV antibiotics were given in the wards, the proportion of oral antibiotics given in the wards was calculated in order to apply nurse preparation costs only for in-hospital treatment.

Material costs for administration of IV treatment were estimated for syringes, needles, peripheral venous catheter, liquids (sodium chloride or sterile water), etc. at SEK15.70. An assumption was made that peripheral venous catheters were changed every fourth day [18].

Indirect cost for production losses are not included since the median age of patients was 83 years [9]. All costs are expressed in SEK in 2015 price level. Unit costs used in the calculations are presented in Table 1. The average exchange rate in 2015 was 1 USD = SEK 8.435 and 1 EUR = SEK 9.356 [19].

**Table 1:** Unit costs of each resource category (in SEK, 2015 price level)

Type of resource	Unit cost	Source
Implementation cost (one-time cost)	13 620.00	Estimated
Hospital stay department of internal medicine (per day)	4 643.00	[26]
Audit with specialist in infectious diseases (per four hours)	2 348.00	[12, 15]
Resident physician in internal medicine (per four hours)	1 536.00	[12, 15]
Materials used per dose of IV treatment	15.70	Assumption
Nurse time (18 min per dose of IV treatment)	95.22	Assumption and [13–15]
Nurse time (6 min per day for oral antibiotics)	31.72	Assumption and [13–15]
IV antibiotics (average cost per day)		
- cefotaxim	23.18	[16]
- bensylpenicillin	36.00	[16]
- cloxacillin	39.90	[16]
- ampicillin	252.00	Personal communication
- piperacillin-tazobactam	43.50	[16]
- imipenem	83.25	[16]
- meropenem	71.70	[16]
Oral antibiotics (average cost per day)		
- phenoxymethylpenicillin	7.11	[17]
- amoxicillin	3.15	[17]
- amoxicillin-calvulanate	15.30	[17]
- dicloxacillin	19.62	[17]
- ciprofloxacin	5.20	[17]
- trimethoprim-sulfamethoxazole	5.65	[17]
- tetracycline	8.35	[17]
- clindamycin	14.73	[17]
- metronidazole	7.10	[17]
- nitrofurantoin	3.51	[17]
- pivmecillinam	23.05	[17]

### Data analysis

The antimicrobial stewardship program was implemented during five months. The costs for the program was calculated for these five months and then extrapolated to one whole year, with the exception of the implementation cost. This extrapolation may not reflect a true yearly cost as monthly costs, as well as effect sizes may be reduced during a longer course of a program. All costs were estimated for two periods, one period when the stewardship program was implemented (2013) and one control period (2012).

Three analyses of costs comparisons were performed (Additional file 1: Table S1). Direct costs for project implementation, for each antibiotic (including material costs) and for utilization of infectious disease physician time were specifically allocated to the project and directly comparable between the two periods. A comparison between the intervention period and control period was made including only these direct costs. This was designated analysis one. Costs for nurse time (for preparation and administration of antibiotics) and ward physician time are opportunity costs not specifically allocated to the project, but directly comparable between the two projects. Opportunity costs in this specific setting means that the nurses and doctors can potentially be freed and be available for other tasks. A second comparison, analysis two, was performed including these costs. The direct costs for hospital-lengths of stay for patients treated with antibiotics in each period were included in analysis three. However, since fewer individuals were started on antibiotics during the stewardship intervention and since there were no statistically significant differences in patient length-of-stay between the two periods [9], it can be debated whether the periods can be considered directly comparable with regards to total lengths of hospital stay. The same reasoning applies to mortality and readmission, as even though the absolute numbers were lower during the stewardship intervention the proportions were the same, and no significant differences in 28-day mortality or 28-day readmissions between the two periods were demonstrated in the original report [9]. Our view as that an inclusion of these outcome terms in the direct cost analysis would not be meaningful, but rather skew the costs in favour of the intervention. Thus, data on mortality and on readmissions were presented (Table 2) but not included in the cost analyses. An attempt to address the indirect or societal costs of the program was not performed.

**Table 2:** Unit or days of utilization per resource category and study period (costs in SEK, 2015 price level)

Type of resource/cost unit^a^	Total number of days/units during stewardship intervention 2013	Total number of days/units during control period 2012
Implementation cost (units)	1	0
Hospital stay (days)	7193	7402
ID specialist time (units)	40	0
Resident physician time (units)	40	0
IV antibiotic treatment, materials (doses)	6472	7251
IV antibiotic treatment, nurse time (doses)	6472	7251
Hospital oral antibiotic treatment, nurse time (days)	1477	2975
Cefotaxim	1373	1882
bensylpenicillin	423	394
cloxacillin	226	202
ampicillin	6	5
piperacillin-tazobactam	270	206
meropenem	35	68
phenoxymethyl-penicillin	810	789
Amoxicillin	1109	1638
dicloxacillin	270	300
Ciprofloxacin	534	1110
trimethoprim-sulfamethoxazole	489	557
Tetracycline	409	794
Clindamycin	219	462
metronidazol	255	221
Nitrofurantoin	86	150
Pivmecillinam	283	427
Death within 28 days (no. of patients)	108	117
Readmission within 28 days (no. of patients)	180	203

^a^All antibiotic costs are in full days

Sensitivity analyses were performed to evaluate the robustness of the base case results for analysis two by varying the most important cost drivers; physician time and nursing time. In addition, the material costs for preparation of IV antibiotics were varied between SEK14 and SEK18 instead of SEK15.70 from the base case analysis. The weekly time for physician audits was varied from eight hours weekly to six hours per week. Nurse time for administration of IV antibiotics was varied between 16 and 20 min per preparation instead of 18 min as in the base case while the time for administration of oral antibiotics was varied between 4 and 8 min instead of 6 min. The sensitivity analyses were confined to analysis two.

## Results

### Cost units during the stewardship and the control period

The implementation process of the program itself demanded 20 h of Infectious Diseases specialist physician time. The total hospital length of stay for all patients was 7 193 days for the stewardship period and 7 402 days for the control period. The total days of oral treatment that were given in-hospital in each cohort was calculated to be 1 477 in the stewardship cohort and 2 975 in the control cohort. Resource utilization for each cost unit and cohort is presented in Table 2.

### Total cost of each unit of the implementation of an antimicrobial stewardship program

For each resource category, the total cost per cohort and the overall balance is presented in Table 3. A negative balance in a resource category implies that the stewardship intervention leads to cost-savings in the actual category. Categories where substantial savings occurred as a result of the stewardship program include the cost of nurse time for administration of IV and oral antibiotics and the material costs of IV antibiotics. Moderate cost-savings were found in several categories of antibiotics (Table 3).

**Table 3:** The total cost of the stewardship intervention per resource category (in SEK, 2015 price level). The total yearly balance in each analysis is bolded

Type of resource/cost unit	Cost during the stewardship intervention	Cost during the control period	Total balance per resource	Included in analysis
Implementation cost^a^	13 620	0	13 620	1,2,3
Hospital stay	33 397 099	34 367 486	−970 387	3
ID specialist time	93 920	0	93 920	1,2,3
Resident physician time	61 440	0	61 440	2,3
IV antibiotic treatment, materials	101 610	113 841	−12 230	1,2,3
IV antibiotic treatment, nurse time	616 264	690 440	−74 176	2,3
Oral antibiotic treatment, nurse time	46 850	94 367	−47 517	2,3
cefotaxim	31 826	43 625	−11 799	1,2,3
bensylpenicillin	15 228	14 184	1 044	1,2,3
cloxacillin	9 017	8 059	958	1,2,3
ampicillin	1 512	1 260	252	1,2,3
piperacillin-tazobactam	11 745	8 961	2 784	1,2,3
meropenem	2 510	4 876	−2 366	1,2,3
phenoxymethyl-penicillin	5 759	5 610	149	1,2,3
amoxicillin	3 493	5 159	−1 666	1,2,3
dicloxacillin	5 298	5 886	−588	1,2,3
ciprofloxacin	2 777	5 772	−2 995	1,2,3
trimethoprim-sulfamethoxazole	2 763	3 147	−384	1,2,3
tetracycline	3 415	6 630	−3 215	1,2,3
clindamycin	3 226	6 805	−3 579	1,2,3
metronidazol	1 810	1 569	241	1,2,3
nitrofurantoin	302	527	−225	1,2,3
pivmecillinam	6 523	9 842	−3 319	1,2,3
Analysis one (yearly cost)	800 948	638 958	**161 990**	
Analysis two (yearly cost)	2 684 569	2 679 456	**5 113**	
Analysis three (yearly cost)	(89 517 026)	(92 034 920)	**(−2 517 894)**	

^a^The implementation cost was a one-time cost, and was not extrapolated in the yearly calculation

### The cost analyses of implementation of the stewardship program

In analysis one, only direct and comparable costs specifically allocated to the stewardship program were included (for resource categories included, see Table 3). In this analysis, the extrapolated yearly cost of the stewardship program was SEK 161 990 (approximately USD 19 205 and EUR 17 313).

In analysis two, direct and comparable costs as well as comparable opportunity costs were included. This allowed the addition of the cost of ward staff time in the evaluation (for included resource categories, see Table 3). In this analysis, the extrapolated yearly cost of the stewardship program was SEK 5 113 (approximately USD 606 and EUR 546).

In analysis three, direct and comparable costs, comparable opportunity costs as well as the costs from the hospital lengths of stay of patients treated with antibiotics were included (for included resource categories, see Table 3). In this analysis, the intervention lead to a lowering of costs, and the extrapolated yearly saving of the stewardship was SEK 2 517 894 (approximately USD 298 506 and EUR 269 115).

The results from the sensitivity analyses are presented in Table 4. Variations on assumptions of the main cost drivers; physician time, nurse time and material costs for analysis two resulted in a total range from cost reduction of SEK 153 574 to a maximum cost of SEK 76 734.

**Table 4:** Results of base case and sensitivity analyses of analysis two, yearly cost (in SEK, 2015 price level)

Type of resource/cost unit	Cost during the stewardship intervention	Cost during the control period	Balance
Base case analysis two	2 684 569	2 679 456	5 113
Physician time 6 h per week	2 583 585	2 679 456	-95 871
Nurse time 16 min for IV antibiotics and 4 min for oral antibiotics	2 465 934	2 398 211	67 723
Nurse time 20 min for IV antibiotics and 8 min for oral antibiotics	2 903 205	2 960 908	-57 703
Material cost IV administration SEK14	2 655 963	2 647 407	8 556
Material cost IV administration SEK18	2 723 272	2 722 817	455

## Discussion

Our antimicrobial stewardship was conducted using an individual audit and direct feedback system involving infectious disease specialists, which would be considered a costly variant. It was introduced among a geriatric patient group mainly prescribed low-cost antibiotics in a setting with no on-going outbreak and low general levels of antimicrobial resistance. Despite this, the project almost fully bears its own costs (an extrapolated yearly cost of SEK 5 113 or USD 606) when a conservative approach was performed, including all comparable direct and opportunity costs objectively attributed to the intervention (analysis two). This analysis neither includes costs of the beneficial patient outcomes (including length of stay in hospital), nor the potential long-term societal benefits of reducing antibiotic misuse/overuse.

The strengths of this study include an individual characterization and follow-up of each patient receiving antibiotics during the intervention and control period, allowing a detailed, correct and comparable economic analysis. Another strength is that the study was conducted with no on-going outbreak, reducing the risks of overestimating the effects and providing more generalizable results. The limitations include the limited follow-up time of the intervention and the quasiexperimental (before-after) design. Another potential limitation is that a follow-up only was performed on patients that actually received antibiotics and not all patients in the ward. Fewer patients in total were started on antibiotics during the intervention, and there was a selection towards more severe infections during the stewardship intervention. This was indicated by significantly higher levels of C-reactive protein (CRP) and a significant reduction in antibiotics only used for less severe infections, such as cystitis, during the intervention [9]. The direct costs related to short-term outcomes such as mortality and readmission were thus not directly comparable between the two cohorts, but would rather skew the cost analysis in favour of the intervention, even though they have been demonstrated using statistical analysis of the proportions to be unchanged following the intervention [9].

In the present work, we have applied three different cost analyses. Whenever they are possible to calculate, it is suggested to include all opportunity costs in the analysis of total antimicrobial stewardship costs [8]. Therefore, we argue that analysis one does not provide enough information to be used. Normally, it is suggested that the total costs related to lengths-of-stay also should be included in the analysis of each cohort, which is performed in analysis three. However, since only patients started on antibiotics are included in our analysis, and since the severities of the infections treated with antibiotics in the two periods differ, the two cohorts are difficult to compare with respect to outcomes including hospital lengths-of stay. Also, the fact that fewer hospitalized patients receive antibiotics will not mean that there are fewer patients in the ward, and will not mean a cost reduction for the ward in absolute terms. Due to these concerns, we believe that analysis three is of least value and is put in parenthesis. Our belief is that the most objective analysis is analysis two. We performed a sensitivity analysis of the costs in analysis two, suggesting that there is uncertainty depending on the assumptions made, but that the variation is below 10 % of the total cost.

Even though guidelines have been formulated [20], antibiotic stewardships can be conducted in several different ways, and these decisions clearly have implications for the economic consequences of the program. There have been a number of examples of stewardship interventions with distinct cost reductions as a consequence [21]. Most of these programs have been conducted in areas with high proportions of antimicrobial resistance and use of high-cost antibiotics. Most cost-saving interventions that were not conducted in such areas have other distinctions; including mainly younger patients [18], a focus on specific problem pathogens [22] or on diagnostic interventions [23]. We believe that the results of our stewardship program, targeting mainly elderly hospitalized patients with underlying diseases in a setting with low rates of antimicrobial resistance and not in response to an outbreak, can be generalizable to hospitals in regions with corresponding levels of antimicrobial resistance. This would be especially true in areas with corresponding population demographics. We also believe that it is probable that such a program would work well in a setting with higher levels of antimicrobial resistance. Also, the use of individual audits between an ID-specialist and a ward physician likely provides substantial secondary gains that are not measurable. There is evidence that ID-specialists can improve patient outcomes and induce cost savings [24], but some of the most important consequences of our program have been the bilateral knowledge gain from inter-disciplinary discussions. Even though a total of 8 h of ID physician time per week were spent on the stewardship intervention, this cost analysis demonstrates that the cost of the program is comparatively low.

## Conclusions

The cost analysis provided in this study shows that an audit-based individual antimicrobial stewardship can bear its own costs, even in a situation with low general levels of antimicrobial resistance. The results show that such programs can be performed at a relatively low cost also in regions with lower proportions of antimicrobial resistance. However, to combat antimicrobial resistance on a larger scale, a multitude of simultaneous measures are needed, tailored to each specific setting [25].

## Abbreviations

ASP, antimicrobial stewardship program; ATC, Anatomical therapeutic chemical classification; EUR, Euro; FASS, Farmaceutiska specialiteter i Sverige (Pharmaceutical specialities in Sweden); ID, infectious diseases; IV, intravenous; SEK, Svensk krona (Swedish crown currency); USD, US dollar

## Additional file

Additional file 1: Table S1. Analyses in the dataset (DOCX 47 kb)
