# Supplementary material for: A cost analysis of introducing an infectious disease specialist-guided antimicrobial stewardship in an area with relatively low prevalence of antimicrobial resistance
Source: BMC Health Serv Res. 2016 Jul 27;16:311. doi: 10.1186/s12913-016-1565-5 (PMC4963928; doi:10.1186/s12913-016-1565-5)
Supplement: Additional file 1: Table S1. — Analyses in the dataset (DOCX 47 kb) [file 12913_2016_1565_MOESM1_ESM.docx]

**Table S1. Analyses in the dataset**

| Analysis | Description |
| --- | --- |
| Analysis one | In analysis one, only direct costs attributed to the intervention were included, including costs for implementation, physician time of the ID specialist, antibiotics and materials for administration of antibiotics. |
| Analysis two | In analysis two, all costs from analysis one were included, with the addition of opportunity costs, i.e. resident physician time as well as nursing time for preparation of antibiotics. |
| Analysis three | In analysis three, all costs from analysis one and two were included, with the addition of costs related to hospital lengths-of-stay for individuals treated with antibiotics. |
